# Multipath interaction network of annexin A2: multifaceted regulation and translational perspectives in neurological diseases

**DOI:** 10.3389/fgene.2026.1916220

**Published:** 2026-09-28

**Authors:** Yong Guan, Ping Gu, Dachuang Guo, Liqiang Huang, Dongye Lian, Caicai Zhang, Jigao Feng, Hao Peng

**Affiliations:** 1 Department of Neurosurgery, Hainan Affiliated Hospital of Hainan Medical University, Haikou, China; 2 Department of Neurosurgery, The Second People’s Hospital of Hainan Province, Wuzhishan, China; 3 Key Laboratory of Tropical Translational Medicine of Ministry of Education and Key Laboratory of Brain Science Research Transformation in Tropical Environment of Hainan Province, School of Basic Medicine and Life Sciences, Hainan Medical University, Haikou, China; 4 Department of Neurosurgery, The Second Affiliated Hospital of Hainan Medical University, Haikou, China

**Keywords:** annexin A2 (ANXA2), inflammation, nervous system disorders, signaling pathways, tumorigenesis

## Abstract

Annexin A2 (Anxa2) is a Ca^2+^-dependent phospholipid-binding protein whose functions are shaped by dynamic membrane association, post-translational regulation, and interactions with diverse molecular partners. Rather than acting as a conventional pathway-specific regulator, Anxa2 appears to operate within a multipath interaction network that links membrane organization, cytoskeletal remodeling, vesicular trafficking, extracellular proteolysis, and cellular stress responses. This Review examines how these interconnected functions contribute to neurological diseases, including Parkinson’s disease, Alzheimer’s disease, ischemic stroke, traumatic brain injury, and glioma. We critically evaluate evidence connecting Anxa2 with Phosphatidylinositol 3-kinase/Protein Kinase B (PI3K/AKT), mitogen-activated protein kinase/extracellular signal-regulated kinase (MAPK/ERK), Nuclear Factor kappa-light-chain-enhancer of activated B cells (NF-κB), and Transforming Growth Factor-beta (TGF-β) related signaling and distinguish direct molecular interactions from pathway-dependent or secondary signaling changes. Current evidence suggests that many Anxa2-associated signaling outputs may arise from changes in membrane trafficking, receptor organization, protein handling, and cellular state rather than from direct control of individual signaling cascades. This network-based view also provides a framework for interpreting apparently divergent effects of Anxa2 across neuronal, glial, endothelial, immune, and tumor contexts. From a translational perspective, Anxa2 is therefore a promising but challenging target: its broad physiological functions argue against indiscriminate inhibition and instead favor disease-, cell-, and interaction-specific modulation. Defining the molecular architecture and context-specific operation of Anxa2-centered networks will be essential for determining its value as a biomarker and therapeutic target in neurological disease.

## 1 Introduction

Annexin A2 (Anxa2), a Ca^2+^-dependent phospholipid-binding protein (Huang et al., 2022), has emerged as an important modulator of membrane dynamics through its ability to associate with phospholipid membranes and interact with diverse molecular partners. Rather than functioning as a classical signaling molecule, Anxa2 is increasingly recognized as a membrane-associated scaffold that coordinates multiple biological processes, including membrane trafficking, cytoskeletal remodeling, fibrinolysis, vesicular transport, and cellular stress responses (Huang et al., 2022; Lim and Hajjar, 2021). Consistent with these diverse functions, Anxa2 has been implicated in a broad spectrum of pathological conditions, including cancer, inflammatory diseases, cerebrovascular disorders, and neurodegenerative diseases. However, the effects attributed to Anxa2 vary considerably across tissues, cell types, disease models, and experimental conditions. Whether this heterogeneity reflects genuine cell-specific functions or differences in experimental context and secondary cellular responses remains unresolved (Madureira and Waisman, 2013). Despite substantial progress in Anxa2 research, several fundamental questions remain unresolved. Although Anxa2 has been reported to be associated with multiple signaling pathways, including PI3K/AKT, MAPK/ERK, NF-κB, and TGF-β-related networks, the underlying molecular mechanisms remain incompletely understood. In many cases, available evidence is primarily based on expression analyses, genetic perturbation, or pathway-associated observations rather than direct mechanistic demonstration. Moreover, intracellular Ca^2+^ signaling regulates numerous biological processes, making it difficult to attribute specific downstream responses solely to Anxa2 activation. Likewise, phenotypes observed following Anxa2 depletion may partly reflect secondary alterations in membrane organization, vesicular trafficking, or cellular homeostasis rather than direct loss of Anxa2-mediated signaling (Gauthier-K et al., 2011; San et al., 2016; Astillero-Lopez et al., 2024; He et al., 2024; Tian et al., 2024). These limitations contribute to the apparent discrepancies reported across different experimental models and highlight the need for a more critical evaluation of the current evidence.

In this Review, we critically examine the structural characteristics, regulatory mechanisms, protein interaction networks, signaling-associated functions, and translational potential of Anxa2, with particular emphasis on neurological diseases. Rather than considering Anxa2 as a universal upstream regulator of multiple signaling pathways, we discuss how its membrane-associated scaffold properties and dynamic protein–protein interactions may contribute to distinct biological responses across cell types and disease settings. We further evaluate current mechanistic limitations, reconcile apparently conflicting findings where possible, and outline key questions that should guide future studies aimed at defining disease-specific functions of Anxa2 and developing more precise therapeutic strategies.

## 2 Structure and function of Anxa2

The annexin family comprises Ca^2+^- and phospholipid-binding proteins with high structural homology that play pivotal roles in Ca^2+^-regulated membrane events, including organelle organization, membrane trafficking, and cytoskeletal interactions (Schloer et al., 2018). As a calcium-modulated protein, Anxa2 exhibits an intricate molecular architecture and multifunctional roles (Huang et al., 2022; Bharadwaj et al., 2013). With a molecular mass of approximately 39 kDa, Anxa2’s core structure comprises multiple distinct domains that facilitate dynamic interactions with plasma membranes and other cellular constituents (Grindheim et al., 2017). Its architecture includes two fundamental domains: (i) a variable N-terminal head domain that mediates binding to diverse ligands and modulates protein functionality, and (ii) a conserved C-terminal core domain, exhibiting high structural conservation across all Anxa2 isoforms, that is primarily responsible for anchoring to endogenous membrane lipids (Huang et al., 2022; Bharadwaj et al., 2013; Grindheim et al., 2017; Bharadwaj et al., 2021). These structural characteristics enable Anxa2 to perform critical roles in membrane trafficking, endocytosis, exocytosis, tissue remodeling, and regulation of Ca^2+^ signaling and cell signaling (Cockrell et al., 2008; Koe et al., 2017; Ma et al., 2018). Anxa2 has also been described as a scaffold protein linking membrane dynamics with cytoskeletal organization (Grieve et al., 2012).

### 2.1 Regulatory mechanisms controlling Anxa2 functional states

Beyond its structural features, Anxa2 activity is regulated by mechanisms that influence its localization, molecular interactions, and functional states. These regulatory processes may contribute to the heterogeneous effects associated with Anxa2 across different experimental and biological settings (Gerke et al., 2024). Calcium-dependent membrane binding represents a key determinant of Anxa2 function. Increased intracellular Ca^2+^ levels can promote conformational changes in the annexin core domain, facilitating phospholipid binding and redistribution of Anxa2 from the cytoplasm to membrane-associated compartments. Through this dynamic localization, Anxa2 participates in membrane remodeling, cytoskeletal organization, vesicular trafficking, and receptor-associated signaling events.

Although intracellular Ca^2+^ elevation represents an important trigger for Anxa2 membrane association, Ca^2+^ influx itself is not sufficient to define a specific Anxa2-mediated biological response. Calcium signaling regulates numerous cellular processes, and the functional consequences of Anxa2 recruitment are likely determined by additional regulatory factors, including membrane composition, post-translational modifications, interacting partners, and subcellular localization. For example, phosphorylation at Tyr23 has been implicated in controlling Anxa2 membrane translocation and extracellular exposure (Gabel et al., 2020; Grindheim et al., 2017), whereas formation of the Anxa2–S100A10 complex contributes to its extracellular and membrane-associated functions (Weijie, 2024). Therefore, Anxa2 activation should be considered a multistep regulatory process rather than a direct consequence of Ca^2+^ elevation alone.

6125In addition to Ca^2+^-dependent membrane recruitment, interaction with S100A10 (p11) represents another important regulatory mechanism that stabilizes Anxa2 localization and enables its extracellular functions (Weijie, 2024). Moreover, different interacting partners may influence whether Anxa2 primarily functions as a scaffold, signaling mediator, or extracellular regulatory factor.

Moreover, genetic depletion studies should be interpreted with caution. Although Anxa2 knockout or knockdown approaches have provided valuable evidence regarding its biological relevance, loss of Anxa2 may indirectly alter broader cellular states, including membrane organization, vesicular trafficking, and inflammatory responses. Therefore, phenotypes observed in these models should be interpreted together with complementary mechanistic approaches.

## 3 Anxa2-associated signaling changes in neurological disorders: evidence strength and alternative mechanisms

Anxa2 has been linked to changes in several signaling networks, including PI3K/AKT, MAPK/ERK, NF-κB, and TGF-β-associated pathways. The strength of these associations, however, varies considerably among studies. In many cases, pathway involvement has been inferred from changes in phosphorylation, transcriptional readouts, or pharmacological inhibition following Anxa2 overexpression or depletion. Such experiments establish that pathway activity changes when Anxa2 is perturbed, but do not necessarily demonstrate that Anxa2 is a pathway-specific upstream regulator. This distinction is particularly important because Anxa2 has established roles in membrane organization, actin remodeling, endocytosis, and endosomal trafficking (Grieve et al., 2012; Gerke et al., 2024; Morel et al., 2009). Perturbing these fundamental processes can alter receptor localization, cargo sorting, membrane integrity, and cellular stress, each of which may secondarily affect multiple signaling pathways. The evidence discussed below is therefore interpreted according to whether it supports a direct molecular interaction, pathway dependence of an Anxa2-associated phenotype, or a downstream association.

### 3.1 Evidence for Anxa2-associated PI3K/AKT signaling in CNS pathology

The PI3K/AKT/mTOR pathway regulates cell survival, metabolism, angiogenesis, and tissue repair and is implicated in neurological injury and neurodegeneration (M et al., 2019; Rai et al., 2019; Glaviano et al., 2023; Xu et al., 2020). Evidence connecting Anxa2 to this pathway in the CNS is concentrated mainly in vascular models (Figure 1).

**FIGURE 1:**
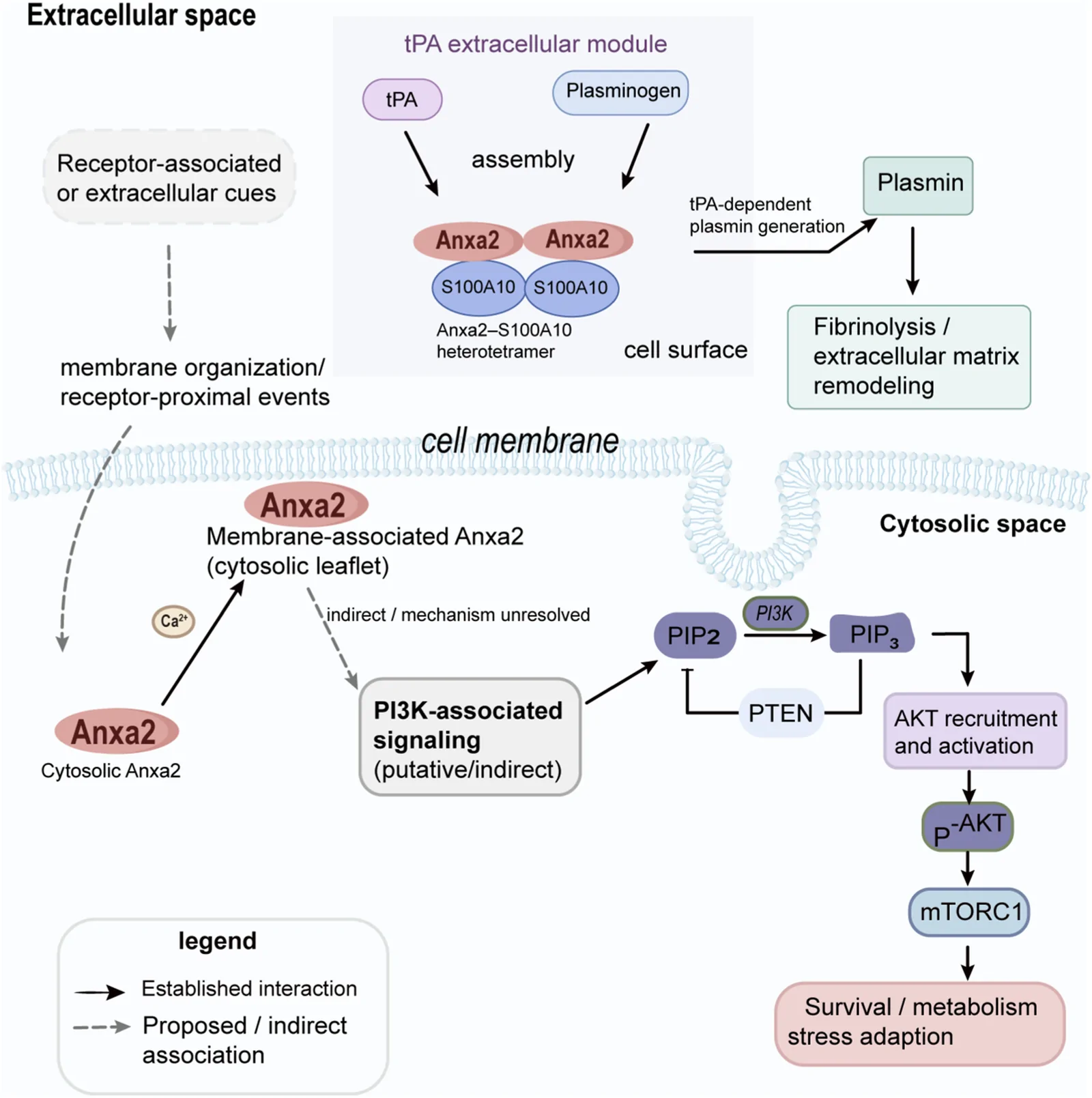
Proposed model of Anxa2-linked extracellular fibrinolysis and PI3K–AKT–mTOR signaling: At the cell surface, the Anxa2–S100A10 heterotetramer provides a platform for the co-assembly of tissue plasminogen activator (tPA) and plasminogen, promoting plasmin generation and extracellular fibrinolysis/extracellular matrix remodeling. On the cytosolic leaflet, Ca^2+^-dependent membrane recruitment of Anxa2 may influence membrane organization and receptor-proximal events linked to PI3K signaling. PI3K converts phosphatidylinositol 4,5-bisphosphate (PIP_2_) to phosphatidylinositol 3,4,5-trisphosphate (PIP_3_), whereas PTEN counteracts this reaction. PIP_3_ promotes AKT recruitment and activation, followed by mTORC1-associated responses involved in cell survival, metabolic adaptation and stress responses. Solid arrows indicate established interactions; dashed arrows indicate proposed or indirect associations.

In experimental stroke, increased Anxa2 expression and recombinant Anxa2 treatment enhanced endothelial migration, tube formation, and post-ischemic angiogenesis. The same study reported an interaction between Anxa2 and the proposed Anxa2 receptor and showed that pharmacological inhibition of AKT or ERK reduced the pro-angiogenic effects of recombinant Anxa2 (Lin et al., 2023). These experiments support a requirement for AKT/ERK activity in the observed endothelial phenotype, but they do not by themselves establish Anxa2 as a direct upstream activator of either kinase. Changes in membrane organization, receptor availability, adhesion, or cytoskeletal dynamics could also influence the same angiogenic readouts.

A similar relationship between Anxa2 expression and PI3K/AKT activity has been reported in retinal endothelial cells and oxygen-induced retinopathy (Li et al., 2022). This finding is relevant to vascular biology but should be extrapolated cautiously to cerebral endothelial cells because retinal and brain vascular beds are not equivalent. Moreover, evidence from non-neural systems is not directionally uniform. In cancer cells, Anxa2 depletion enhanced AKT activation after EGF or oncogenic Ras stimulation, and Anxa2 was reported to interact with PTEN through a redox-sensitive mechanism (Castaldo et al., 2019). Thus, Anxa2 perturbation can be associated with either increased or decreased AKT activity depending on the experimental system.

More direct mechanistic evidence has recently emerged from a diabetic cerebral ischemia–reperfusion model, in which phosphorylated Anxa2 was reported to interact with Akt in mitochondria and to influence mitochondrial apoptosis (Zhou et al., 2025). Although this study provides stronger evidence for a physical Anxa2–Akt relationship, the mechanism was examined in a specific metabolic and endothelial context and should not yet be generalized to other CNS cell types.

Taken together, current data support an association between Anxa2-dependent cellular states and PI3K/AKT activity, particularly in vascular injury. They do not support a universal Anxa2→PI3K/AKT signaling hierarchy. Future studies will need to distinguish kinase regulation from secondary effects of altered membrane trafficking, receptor organization, and cell state.

### 3.2 MAPK/ERK signaling: Pathway dependence does not establish Anxa2 specificity

The MAPK/ERK pathway is a critical signaling cascade involved in cellular proliferation, survival, migration, and tissue remodeling (Park, 2023). Evidence linking Anxa2 to MAPK/ERK signaling overlaps substantially with that described for angiogenesis (Figure 2). In the ischemic brain, AKT/ERK inhibition attenuated recombinant Anxa2-induced endothelial migration and tube formation (Lin et al., 2023), indicating that these kinases participate in the angiogenic response. Whether Anxa2 directly controls ERK activation remains unresolved.

**FIGURE 2:**
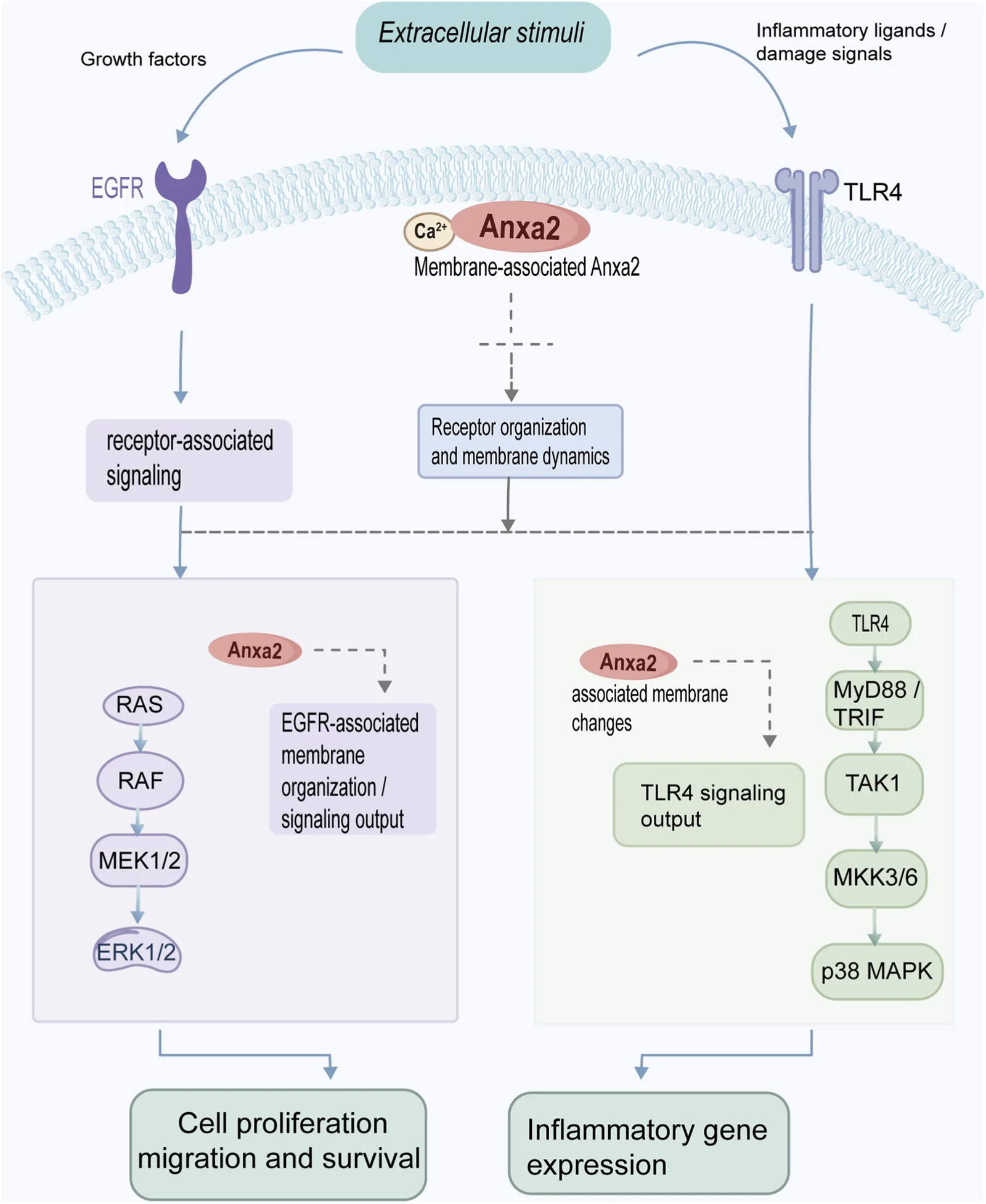
Proposed model of Anxa2-associated modulation of MAPK signaling: Extracellular cues, including growth factors and inflammatory or damage-associated signals, activate membrane receptors such as EGFR and TLR4 and promote Ca^2+^-dependent membrane association of Anxa2. Anxa2-associated changes in membrane organization may modulate receptor-proximal signaling output. In the EGFR branch, signaling proceeds through RAS, RAF, MEK1/2 and ERK1/2 and is associated with cell proliferation, migration and survival. In the TLR4 branch, signaling proceeds mainly through MyD88/TRIF, TAK1, MKK3/6 and p38 MAPK and is associated with inflammatory gene expression. Dashed arrows denote proposed modulation rather than direct molecular binding.

The glioma literature also requires careful distinction between Anxa2-dependent phenotypes and MAPK-specific mechanisms. Anxa2 knockdown reduces glioma cell migration, invasion, angiogenesis, and tumor growth, but these effects have been linked in part to extracellular plasmin activity and membrane-associated functions rather than specifically to ERK signaling (Zhai et al., 2011; Matsumoto et al., 2020). Another study identified an Anxa2–STAT3–oncostatin M receptor axis that promotes mesenchymal features in glioblastoma (Matsumoto et al., 2020), providing evidence for an Anxa2-associated signaling program but not for direct MAPK/ERK regulation. Similarly, changes in cell survival associated with Anxa2-dependent membrane repair do not establish MAPK signaling as the primary mechanism (San et al., 2016).

These observations illustrate an important interpretative limitation. Migration, proliferation, and survival are common endpoints of MAPK activation, but they are also highly sensitive to altered receptor trafficking, cell adhesion, membrane repair, and cytoskeletal remodeling. Consequently, changes in ERK phosphorylation after Anxa2 perturbation should not be considered sufficient evidence for an Anxa2-specific MAPK regulatory axis unless receptor-proximal events and direct molecular requirements are demonstrated.

### 3.3 NF-κb-associated responses: Comparatively stronger evidence with remaining mechanistic uncertainty

Among the pathways discussed here, the association between Anxa2 and NF-κB in CNS inflammation is supported by comparatively more direct experimental evidence (Figure 3). NF-κB is a major regulator of inflammatory gene expression and immune activation (Yu et al., 2020; Guo et al., 2024). In cerebral ischemia–reperfusion models, multi-omics profiling identified increased Anxa2 in activated microglia, while stable Anxa2 knockdown in BV2 cells reduced p65 nuclear translocation, NF-κB transcriptional activity, and expression of TNF-α, IL-1β, and IL-6 after oxygen-glucose deprivation/reoxygenation (Tian et al., 2024). Conditioned medium from Anxa2-depleted microglia also reduced neuronal injury *in vitro*.

**FIGURE 3:**
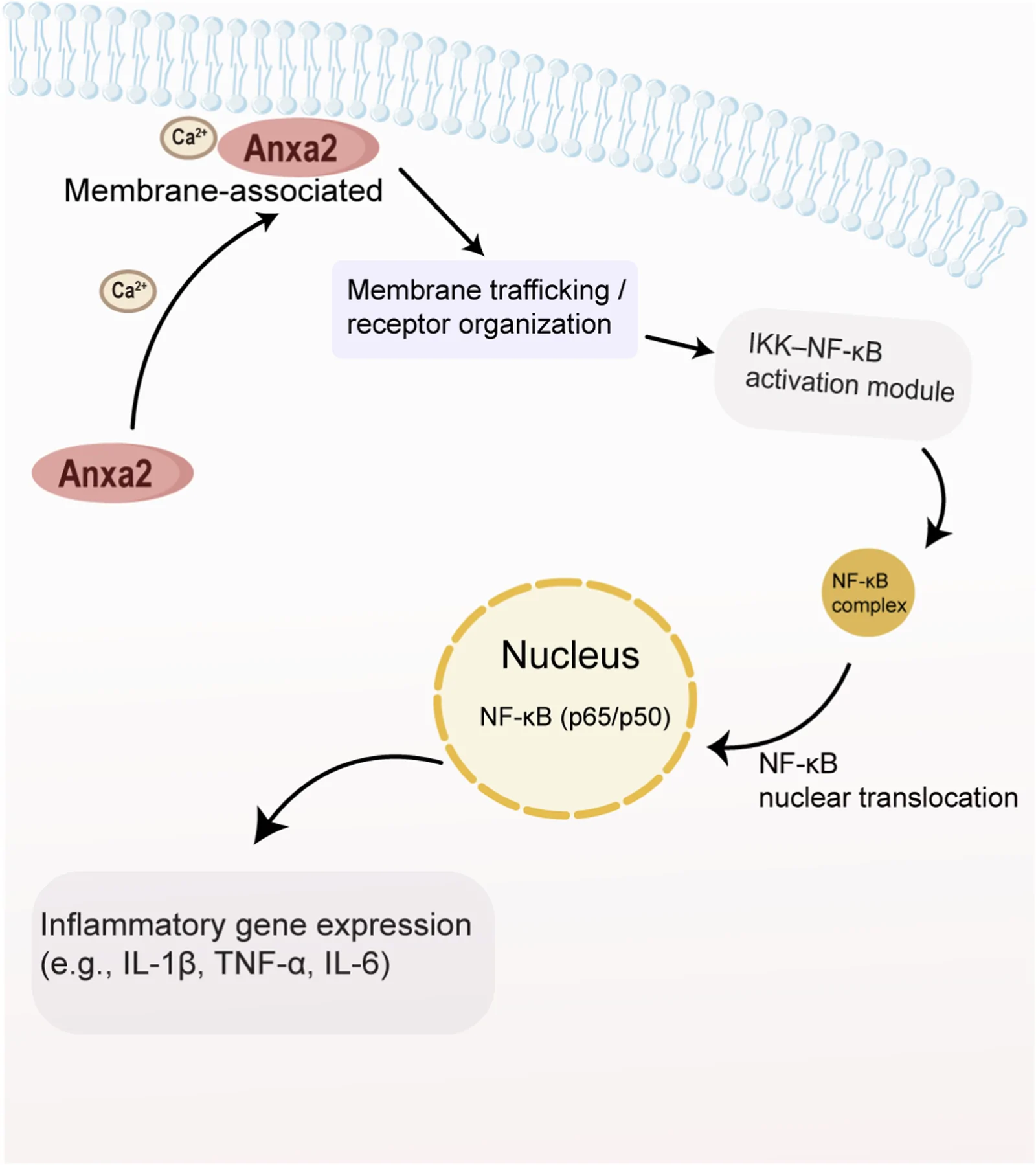
Proposed model of Anxa2-associated modulation of NF-κB responses: Ca^2+^-dependent membrane recruitment of Anxa2 may influence membrane trafficking, receptor organization and inflammatory signaling modules. These changes are proposed to modulate NF-κB activation, including IKK-associated signaling events, resulting in nuclear translocation of NF-κB (p65/p50) and subsequent transcription of inflammatory mediators such as IL-1β, TNF-α and IL-6. Dashed arrows indicate putative or indirect associations.

These findings establish a functional relationship between Anxa2 abundance and NF-κB output in this experimental setting. Nevertheless, the experiments do not fully resolve where Anxa2 enters the signaling sequence. Stable depletion of a membrane- and trafficking-associated protein can alter receptor distribution, endosomal signaling, cytoskeletal organization, or the basal activation state of microglia before NF-κB is measured. Rescue experiments using localization- or interaction-selective Anxa2 mutants would therefore be valuable for separating an NF-κB-specific mechanism from broader changes in microglial physiology.

Evidence from glioblastoma provides additional but biologically distinct support. Stabilization of Anxa2 within circADAMTS6- or S100A11-associated networks has been linked to NF-κB activity and malignant phenotypes (Zhao et al., 2023; Tu et al., 2019). These studies demonstrate that Anxa2 can participate in NF-κB-related regulatory networks in tumor cells, but they should not be treated as direct evidence for the same mechanism in microglia or neurons. Likewise, recombinant Anxa2 reduces leukocyte activation and inflammatory responses after traumatic brain injury (Liu et al., 2021; Cheng et al., 2021), whereas Anxa2 deficiency worsens neurovascular inflammation (Liu et al., 2019). These observations are consistent with an immunomodulatory role but do not define a single cell-autonomous NF-κB mechanism.

Apparent pro- and anti-inflammatory effects of Anxa2 should therefore not automatically be interpreted as intrinsic biological duality. Differences in cell type, extracellular versus intracellular Anxa2, experimental timing, injury model, and the form of perturbation are plausible alternative explanations (Fan et al., 2024).

### 3.4 TGF-β-associated glial responses: An unresolved hypothesis rather than an established signaling axis

TGF-β signaling regulates glial responses, immune homeostasis, and tissue remodeling through canonical Smad-dependent and noncanonical pathways (Deng et al., 2024). Astrocytes display considerable phenotypic heterogeneity across neurological diseases, and their responses are influenced by inflammatory signals and changes in the extracellular environment (Yuan et al., 2023; Kim et al., 2024; Huber et al., 2024; Escartin et al., 2021).

At present, however, direct evidence linking Anxa2 to canonical TGF-β/Smad signaling in CNS cells is limited. Proteomic analysis of the blood–brain barrier in experimental autoimmune encephalomyelitis identified coordinated changes in Anxa2 and several proteins associated with endothelial adhesion and barrier remodeling (Tezuka et al., 2022), but this does not demonstrate that Anxa2 regulates TGF-β receptors or Smad activation. Similarly, studies of Anxa2-related extracellular vesicles or cerebrovascular protection provide evidence for effects on cellular communication and vascular function (He et al., 2024; Fan et al., 2017), not for a defined Anxa2–TGF-β signaling pathway.

Accordingly, the relationship between Anxa2 and TGF-β-associated responses should be regarded as a hypothesis generated from overlapping biological functions rather than as an established molecular axis. Demonstration of Anxa2-dependent changes in TGF-β receptor organization, Smad phosphorylation, or transcriptional output, together with appropriate rescue experiments, will be required before a direct regulatory relationship can be proposed.

### 3.5 Trafficking and membrane organization as alternative explanations for pathway-associated phenotypes

A central limitation of the current literature is that Anxa2 perturbation can affect basic cellular processes upstream of many conventional signaling readouts. Anxa2 contributes to actin-dependent endosome biogenesis, membrane trafficking, cytoskeletal organization, and membrane repair (Grieve et al., 2012; Gerke et al., 2024; Rudenok et al., 2022). Loss of these functions can change the abundance and localization of cell-surface receptors, endosomal residence time, extracellular cargo uptake, membrane tension, and cellular stress. Any of these changes could alter AKT, ERK, NF-κB, or other signaling outputs without requiring Anxa2 to interact directly with the pathway itself.

This issue is particularly important when interpreting knockout, stable knockdown, or prolonged overexpression experiments. Chronic perturbation allows compensatory remodeling of membrane systems, metabolism, and transcriptional programs. A pathway change measured after such adaptation may therefore represent a secondary cellular state rather than the primary molecular consequence of Anxa2 loss.

The same caution applies to apparently opposing effects across neurological models. For example, Anxa2 depletion reduces inflammatory NF-κB responses in cultured microglia after ischemic stress (Tian et al., 2024; Shu et al., 2026), whereas increased endothelial Anxa2 is associated with post-ischemic angiogenesis (Lin et al., 2023). In astrocytes carrying pathogenic LRRK2, reduced Anxa2 is linked to impaired internalization and endolysosomal handling of extracellular α-synuclein (Escartin et al., 2021). More recently, pharmacological Anxa2 inhibition in humanized and wild-type mouse stroke models was associated with reduced T-cell infiltration and neuroinflammation (Zhang et al., 2025). These findings arise from different cell populations, perturbations, and experimental endpoints and therefore cannot yet be taken as evidence that Anxa2 itself switches between fixed protective and pathogenic states.

For this reason, “context dependence” should be treated as a working interpretation rather than a mechanistic explanation. Before invoking intrinsic functional duality, future studies should test potential hidden variables, including cell identity, disease stage, subcellular localization, extracellular versus intracellular Anxa2, perturbation duration, and compensatory changes in membrane trafficking.

Mechanistically informative approaches would include acute protein depletion, cell-type-specific manipulation, rescue with domain- or localization-selective mutants, and experiments that uncouple Anxa2-dependent trafficking from individual signaling interactions. Such designs should help determine whether a pathway is directly regulated by Anxa2 or simply reports a broader change in cellular organization.

### 3.6 Protein–protein interactions: Distinguishing physical association from pathway causality

The diverse functions attributed to Anxa2 are more convincingly supported by its physical interaction network than by a model in which it acts as a universal pathway regulator (Figure 4). The Anxa2–S100A10 heterotetramer is well characterized and contributes to membrane localization, plasminogen activation, fibrinolysis, and extracellular matrix remodeling (Weijie, 2024; Li et al., 2021a). These biochemical functions provide a direct mechanistic basis for several vascular and extracellular effects of Anxa2.

**FIGURE 4:**
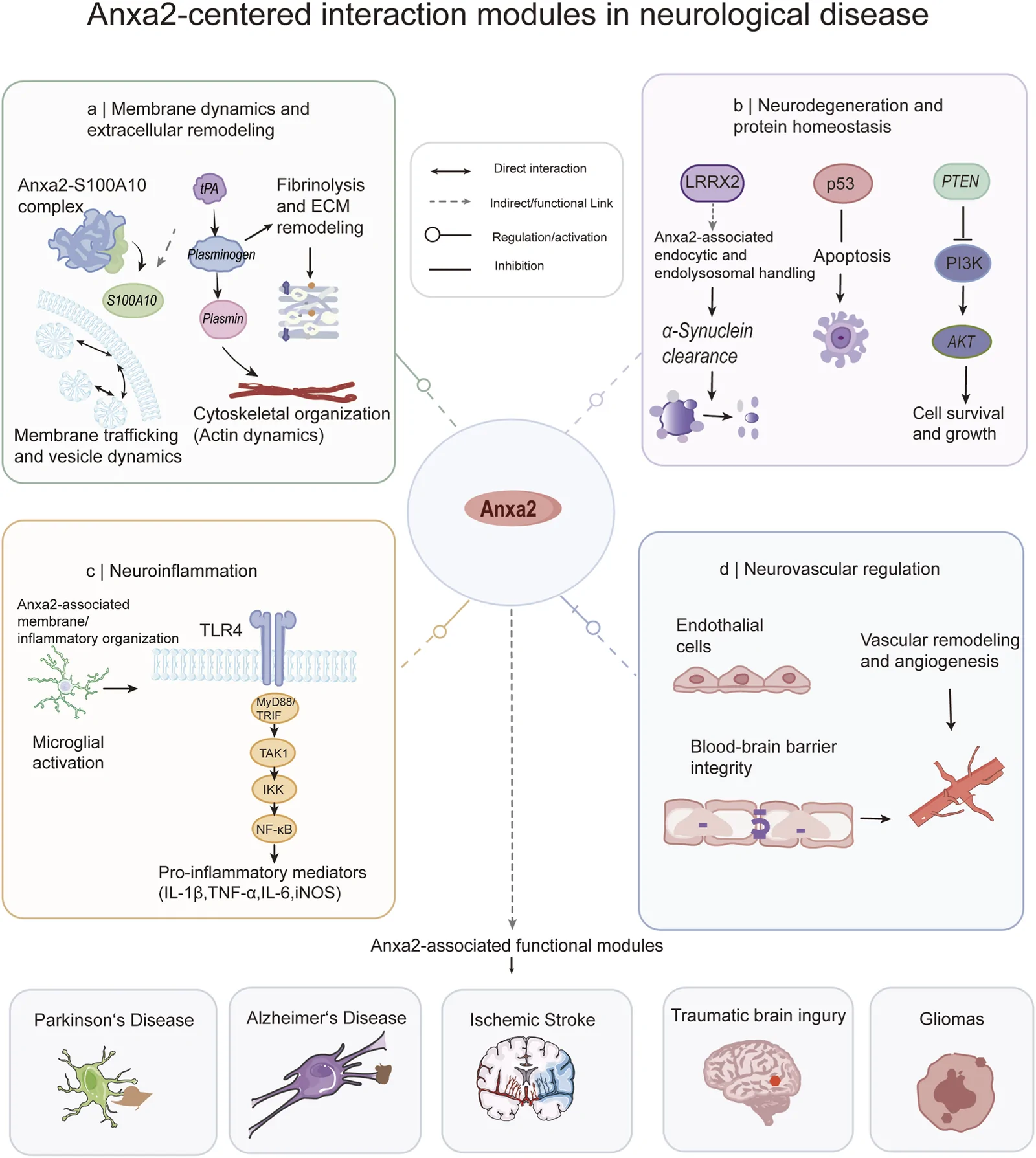
Anxa2-centered interaction modules in neurological disease: **(a)** Membrane dynamics and extracellular remodeling. The Anxa2–S100A10 complex contributes to membrane trafficking and vesicle dynamics and provides a platform for tPA-dependent plasminogen activation, promoting plasmin generation, fibrinolysis and extracellular matrix remodeling. **(b)** Neurodegeneration and protein homeostasis. Reported Anxa2-associated processes include LRRK2-linked endocytic and endolysosomal handling of extracellular α-synuclein, p53-associated apoptosis, and PTEN–PI3K–AKT-related survival signaling. **(c)** Neuroinflammation. Anxa2-associated membrane organization may influence TLR4–MyD88/TRIF–TAK1–IKK–NF-κB signaling and microglial inflammatory output. **(d)** Neurovascular regulation. Anxa2 is linked to endothelial cell function, blood–brain barrier integrity, and vascular remodeling/angiogenesis. Together, these modules may contribute to the pathogenesis of Parkinson’s disease, Alzheimer’s disease, ischemic stroke, traumatic brain injury and glioma. Solid arrows indicate direct or well-established pathway relationships; dashed arrows indicate indirect or functional links.

Other interactions require more restricted interpretation. Anxa2 binding to PTEN has been demonstrated in cancer cells and can alter AKT activity under oxidative signaling conditions (Flood and Hajjar, 2011), but this mechanism has not been established broadly in CNS cells. In Parkinson’s disease models, the relationship between LRRK2 and Anxa2 is supported by proteomic and functional experiments showing that Anxa2 loss impairs astrocytic uptake of extracellular α-synuclein (Escartin et al., 2021; Streubel-Gallasch et al., 2021). Importantly, this phenotype is closely linked to endocytic and endolysosomal function and therefore supports a trafficking mechanism rather than a conventional signaling cascade. Evidence relating Anxa2 to p53-dependent dopaminergic cell death is less definitive: the reported association derives largely from MPP + -treated PC12 cells in which Anxa2 degradation accompanies increased p53 expression and apoptosis (Partevian et al., 2025). This model does not establish a direct Anxa2–p53 regulatory mechanism in dopaminergic neurons *in vivo*.

Likewise, Anxa2-associated regulation of TLR4/NF-κB signaling has been observed in injury and inflammatory models (Liu et al., 2021; Cheng et al., 2021; Castaldo et al., 2019), but changes in receptor trafficking or leukocyte–endothelial interactions remain possible intermediates. Co-immunoprecipitation, expression correlation, or pathway inhibition can establish physical association or functional dependence, but they do not alone define the direction or specificity of a signaling hierarchy.

Overall, the interaction network is best viewed as a set of experimentally supported molecular modules with different levels of mechanistic confidence. Future work should distinguish direct binding events from secondary signaling changes and determine which interactions remain functional at endogenous protein levels in disease-relevant CNS cell types.

## 4 Role of Anxa2 in the central nervous system (CNS)

### 4.1 Anxa2 in neurodegenerative diseases

#### 4.1.1 Parkinson’s disease

Evidence linking Anxa2 to Parkinson’s disease remains heterogeneous and should be interpreted according to the experimental model. In MPP + -treated PC12 cells, reduced Anxa2 expression accompanies increased p53 expression, mitochondrial dysfunction, and apoptosis (Li et al., 2021a). Although these findings suggest an association between Anxa2 loss and cellular vulnerability, the study does not establish that Anxa2 directly regulates p53 in dopaminergic neurons *in vivo*.

More mechanistically informative evidence comes from astrocytic models of LRRK2-associated disease. Astrocytes carrying pathogenic LRRK2 show reduced Anxa2 abundance and impaired uptake and endolysosomal handling of extracellular α-synuclein. Acute Anxa2 knockdown similarly decreases α-synuclein internalization (Streubel-Gallasch et al., 2021; Partevian et al., 2025). These observations support a role for Anxa2 in astrocytic proteostasis, but the most parsimonious interpretation is linked to its established functions in actin-dependent endocytosis and endosomal trafficking rather than to a distinct Anxa2-specific signaling pathway (Morel et al., 2009). Altered Anxa2 expression in MPTP-treated mice has also been reported at early disease stages (Rudenok et al., 2022), although expression changes alone cannot establish whether Anxa2 is causal, compensatory, or simply a marker of altered transport activity.

Thus, current PD data support a role for Anxa2 in cellular transport and protein handling more strongly than they support a direct neuronal signaling mechanism. Whether these trafficking effects ultimately protect against or contribute to α-synuclein pathology will require cell-type-specific studies at endogenous Anxa2 levels.

#### 4.1.2 Alzheimer’s disease

Evidence implicating Anxa2 in Alzheimer’s disease ranges from human association studies to cellular perturbation experiments, but direct causality remains uncertain. Integrative transcriptomic analyses and experiments in Aβ42-producing SH-SY5Y cells identified broad changes in oxidative phosphorylation, endoplasmic reticulum protein processing, SNARE-dependent vesicular transport, autophagy, inflammatory responses, and cell survival after Anxa2 manipulation (Ye et al., 2024). Because several of these processes depend on membrane trafficking and general cellular homeostasis, the resulting phenotypes cannot be assigned to a single Anxa2-specific signaling mechanism.

The relationship between Anxa2 and tau pathology also requires cautious interpretation. In neuronal cells, FLNA overexpression increased tau accumulation and phosphorylation and was accompanied by accumulation of the tau-interacting protein Anxa2 (Levert et al., 2023). These data support a relationship among cytoskeletal organization, Anxa2, and tau localization, but they do not demonstrate that Anxa2 directly controls tau phosphorylation. Similarly, identification of Anxa2 as a hippocampal hub gene by WGCNA provides association rather than mechanistic evidence (Chen et al., 2024).

Human entorhinal cortex proteomics provides complementary pathological evidence: Anxa2 is increased in AD tissue and colocalizes with activated glia and Aβ-containing structures (Astillero-Lopez et al., 2024). Such spatial association is informative but cannot determine whether Anxa2 promotes pathology, participates in aggregate clearance, or reflects reactive glial changes. Together with evidence that Anxa2 influences endothelial barrier properties (Li et al., 2019; Soto-Rojas et al., 2021), these studies place Anxa2 at the intersection of trafficking, glial responses, and neurovascular function. At present, however, its role is better described as disease-associated than disease-driving.

### 4.2 Anxa2 in cerebrovascular and neuroinflammatory disorders

#### 4.2.1 Ischemic stroke

Evidence for Anxa2 in ischemic stroke illustrates why cell type and perturbation strategy need to be separated from presumed “stage-dependent” effects. In endothelial models, increased or recombinant Anxa2 promotes angiogenic responses and is associated with AKT/ERK activity (Lin et al., 2023),while Anxa2 also contributes to endothelial barrier regulation and fibrinolysis through Robo4-associated signaling and the Anxa2/S100A10/tPA system (Flood and Hajjar, 2011; Li et al., 2019). These findings support vascular functions of Anxa2 during ischemic injury.

By contrast, Anxa2 depletion in microglial cells reduces NF-κB-associated inflammatory responses after ischemia–reperfusion (Tian et al., 2024), and a recent humanized T-cell study reported that pharmacological Anxa2 inhibition reduced T-cell infiltration, inflammatory mediator expression, and ischemic injury (Zhang et al., 2025). Additional studies have identified more specific molecular relationships, including mitochondrial Anxa2–Akt interactions in diabetic cerebral ischemia–reperfusion and an interaction between endothelial H3 receptors and Anxa2 during post-ischemic angiogenesis (Zhou et al., 2025; Fan et al., 2022).

These apparently divergent findings should not yet be interpreted as proof that Anxa2 switches from a pathogenic role during acute injury to a reparative role during recovery. The relevant studies examine different cell types, disease models, time points, and forms of Anxa2 manipulation. Cell identity and perturbation strategy therefore represent plausible hidden variables. Demonstrating a true temporal switch will require longitudinal, cell-type-specific manipulation of Anxa2 within the same experimental system.

#### 4.2.2 Traumatic brain injury

Following traumatic brain injury (TBI), Anxa2 has been implicated in regulating neuroinflammatory responses and neurovascular injury. Administration of recombinant Anxa2 (rA2) shortly after TBI was shown to reduce peripheral leukocyte activation and cerebral infiltration, particularly neutrophil recruitment, through suppression of TLR4/NF-κB signaling (Liu et al., 2021; Cheng et al., 2021). Consistently, Anxa2 deficiency was associated with exacerbated early neurovascular inflammation and worsened neurological outcomes following TBI (Liu et al., 2019). However, current evidence is largely derived from experimental models, and the therapeutic relevance of Anxa2 modulation in human TBI requires further validation.

### 4.3 Anxa2 in gliomas

Beyond its involvement in neurological injury and neurodegeneration, Anxa2 is closely associated with glioma progression and tumor-associated inflammatory regulation. Anxa2 has been reported to participate in extracellular vesicle-mediated communication by interacting with heparan sulfate, thereby facilitating tumor cell proliferation and invasive behavior (Ma et al., 2021; Song et al., 2023). Moreover, Anxa2 has been associated with glioma cell migration and invasion through ERK1/2 -associated signaling alterations and regulation of plasma membrane dynamics (Liu et al., 2025; Ham et al., 2023). Clinical studies have demonstrated that elevated Anxa2 expression is associated with shorter overall survival and altered tumor immune microenvironment characteristics in glioblastoma (GBM) patients (Wen et al., 2025). Furthermore, interaction between Anxa2 and Jagged1 intracellular domain (JICD1) has been linked to enhanced tumor stemness and poor prognosis (Wu et al., 2019). Anxa2 has also been identified as a potential target of bufalin, which induces mitochondrial dysfunction and apoptosis in glioma cells, suggesting possible therapeutic implications (Li et al., 2021b). However, most evidence regarding Anxa2 in glioma is derived from cellular and animal models, and its direct contribution as a therapeutic target remains to be established.

Across neurological disorders, Anxa2 has been associated with protein homeostasis, neuroinflammation, vascular remodeling, and tumor-related processes (Table 1). The direction and magnitude of these associations vary across studies, but this heterogeneity should not be interpreted as evidence of intrinsic functional duality. Differences in cell identity, disease model, experimental timing, subcellular localization, and the mode of Anxa2 perturbation may contribute to the divergent findings. In neurodegenerative diseases such as Parkinson’s and Alzheimer’s disease, Anxa2 has been associated with both detrimental and protective effects, including regulation of protein aggregation, autophagy–lysosomal function, oxidative stress, and glial activation, likely reflecting differences in experimental models and disease stages.

**TABLE 1:** Functional mechanisms and molecular determinants of Anxa2 in neurological disorders.

Disease context	Major biological processes	Functional role of Anxa2	Mechanistic pathways and molecular determinants	Evidence limitations	References
Parkinson’s disease (PD)	α-synuclein homeostasis; neuronal survival; neuroinflammation; oxidative stress; vesicular trafficking	Membrane-associated scaffold linked to proteostasis and cellular stress responses	Apoptotic regulation associated with p53 signaling; α-synuclein trafficking involving LRRK2-associated interactions; autophagy–lysosomal regulation	Reported effects differ among experimental models; causal direction, cell-type specificity, and endogenous mechanisms remain incompletely defined	(Morel et al., 2009) (Rudenok et al., 2022) (Li et al., 2021a) (Streubel-Gallasch et al., 2021; Partevian et al., 2025)
Alzheimer’s disease (AD)	Tau pathology; Aβ metabolism; autophagy; synaptic dysfunction; neurovascular alterations	Potential modulator of protein processing and membrane trafficking; candidate disease-associated marker	Tau-related protein processing; SNARE-mediated vesicular trafficking; oxidative stress regulation; autophagy-associated pathways involving HSP90AA1/PTK2B-associated networks	Whether Anxa2 directly drives AD pathology or reflects disease-associated changes remains unclear	(Ye et al., 2024) (Levert et al., 2023) (Chen et al., 2024) (Astillero-Lopez et al., 2024) (Li et al., 2019; Soto-Rojas et al., 2021)
Ischemic stroke	Angiogenesis; BBB repair; fibrinolysis; inflammation; vascular remodeling	Endothelial-associated scaffold and regulator of vascular responses	Anxa2/S100A10/tPA-associated fibrinolytic regulation; AKT/ERK-associated angiogenic responses NF-κB-related inflammatory regulation; Robo4-associated endothelial signaling	Reported effects vary across cell types, disease models, experimental timing, and perturbation strategies; a true stage-dependent functional switch has not been established	(Lin et al., 2023; Zhou et al., 2025; Zhang et al., 2025; Flood and Hajjar, 2011; Li et al., 2019; Tian et al., 2024; Fan et al., 2022)
Traumatic brain injury (TBI)	Neuroinflammation; leukocyte infiltration; neurovascular injury	Immunomodulatory mediator regulating inflammatory responses	TLR4/NF-κB-associated inflammatory regulation; modulation of immune cell recruitment	Evidence is mainly based on experimental models; therapeutic relevance requires further validation	(Liu et al., 2021; Cheng et al., 2021; Liu et al., 2019)
Glioma	Tumor invasion; migration; stemness; immune microenvironment remodeling	Tumor-associated scaffold and potential prognostic biomarker	ERK1/2-associated migration signaling; extracellular vesicle-mediated communication; Jagged1/JICD1- and S100A11-associated regulatory networks	Most evidence derives from cell and animal models; direct therapeutic efficacy remains to be established	(Ma et al., 2021; Song et al., 2023; Liu et al., 2025; Ham et al., 2023; Wen et al., 2025; Wu et al., 2019; Li et al., 2021b)

In cerebrovascular disorders, Anxa2 is linked to vascular repair, endothelial barrier integrity, fibrinolysis, and inflammatory regulation, although it remains unclear whether these effects arise from direct signaling roles or from its function as a membrane-associated scaffold. A similar uncertainty exists in gliomas, where Anxa2 correlates with tumor progression and poor prognosis, but current evidence is largely derived from *in vitro* and animal studies, limiting causal interpretation and clinical translation.

Collectively, the neurological literature supports the involvement of Anxa2 in membrane trafficking, protein handling, neurovascular regulation, inflammatory responses, and tumor-associated processes. The evidence does not yet justify a single model in which Anxa2 directly controls multiple canonical signaling pathways. Nor should apparently opposing findings automatically be interpreted as intrinsic functional duality. Differences among studies may arise from distinct cell populations, disease models, perturbation methods, subcellular localization, and secondary changes in membrane trafficking or cellular homeostasis.

Accordingly, Anxa2 is best considered a multifunctional membrane-associated protein whose perturbation can reshape several cellular processes that converge on conventional signaling readouts. Determining which effects are Anxa2-specific will require experiments that separate its trafficking and structural functions from discrete protein interactions and signaling events.

## 5 Translational perspectives and therapeutic challenges of Anxa2 in neurological disorders

The multifunctional properties of Anxa2, including its ability to regulate membrane dynamics, extracellular interactions, vesicular trafficking, and inflammatory responses, have attracted increasing interest as a potential therapeutic target in neurological disorders. However, unlike conventional disease-driving proteins, Anxa2 participates in both pathological and physiological processes, including membrane repair, vascular homeostasis, fibrinolysis, and cellular stress adaptation (Huang et al., 2022); (Weijie, 2024); (Flood and Hajjar, 2011). Therefore, therapeutic strategies targeting Anxa2 require careful consideration of disease context, cellular specificity, and temporal regulation rather than simple inhibition of total Anxa2 expression.

In cerebrovascular disorders, Anxa2-associated pathways represent one of the most extensively investigated translational directions. Experimental studies of ischemic stroke have associated Anxa2 with endothelial migration, angiogenic remodeling, and vascular recovery, with AKT/ERK activity accompanying some of these vascular phenotypes (Lin et al., 2023). Whether these effects reflect direct pathway regulation or broader changes in membrane organization and endothelial function remains unresolved. In addition, the Anxa2/S100A10/tissue plasminogen activator (tPA) complex regulates plasmin generation and extracellular fibrinolysis, thereby influencing vascular integrity and tissue remodeling after injury (Flood and Hajjar, 2011). These findings suggest that enhancing specific Anxa2-associated vascular functions may provide therapeutic benefits during the recovery phase of ischemic injury. However, because Anxa2 also participates in acute inflammatory responses through microglial activation and NF-κB-associated signaling, herapeutic strategies will require cell-specific and temporally resolved validation rather than assuming a fixed stage-dependent effect of Anxa2 (Tian et al., 2024).

Beyond vascular disorders, targeting Anxa2-associated inflammatory pathways represents another potential therapeutic avenue. In traumatic brain injury, recombinant Anxa2 administration has been shown to reduce leukocyte activation and neuroinflammatory responses through regulation of TLR4/NF-κB signaling, whereas Anxa2 deficiency exacerbates inflammatory damage and neurological deficits in experimental models (Liu et al., 2021; Cheng et al., 2021; Liu et al., 2019). These observations indicate that Anxa2 may function as an endogenous regulator of immune responses rather than simply a pro-inflammatory mediator. Nevertheless, whether modulation of Anxa2 activity can achieve clinically meaningful neuroprotection remains unclear, particularly because inflammatory responses have both detrimental and reparative roles during CNS injury.

In neurodegenerative diseases, therapeutic exploration of Anxa2 remains at an early stage but provides several mechanistic opportunities. In Parkinson’s disease, modulation of Anxa2-associated protein trafficking and lysosomal pathways may influence α-synuclein clearance through interactions involving LRRK2 and vesicular transport mechanisms (Li et al., 2021a; Streubel-Gallasch et al., 2021; Partevian et al., 2025). Similarly, emerging evidence suggests that Anxa2 contributes to Alzheimer’s disease-associated processes, including protein homeostasis, oxidative stress regulation, vesicular trafficking, and neurovascular dysfunction (Ye et al., 2024; Li et al., 2019); (Astillero-Lopez et al., 2024). However, current evidence remains insufficient to determine whether altered Anxa2 expression represents a primary pathogenic mechanism or a compensatory response to neuronal stress. Therefore, future therapeutic strategies should focus on regulating specific Anxa2-mediated molecular interactions rather than globally suppressing Anxa2 activity.

A major challenge for Anxa2-based intervention is its extensive physiological involvement across multiple cell types. Systemic inhibition of Anxa2 may disrupt essential processes, including membrane repair, endothelial barrier maintenance, extracellular matrix remodeling, and intracellular trafficking (Weijie, 2024) (Flood and Hajjar, 2011). This limitation is particularly relevant in neurological diseases, where neuronal, astrocytic, microglial, and endothelial populations may exhibit distinct Anxa2 functions. Consequently, cell-type-specific delivery systems, interaction-domain targeting, and disease-stage-dependent modulation may represent more feasible approaches than conventional pharmacological inhibition.

Recent advances in molecular delivery technologies and regenerative medicine provide additional opportunities for refining Anxa2-targeted strategies. Mesenchymal stem/stromal cells (MSCs) and their extracellular vesicles have demonstrated immunomodulatory and tissue-repair properties that overlap with several biological processes regulated by Anxa2, including inflammatory regulation, vascular repair, and extracellular communication (Khalilzad et al., 2025; Zhou and Shi, 2023); (Yang et al., 2023). However, direct regulatory relationships between MSC-based therapies and Anxa2 remain insufficiently established, and such approaches should currently be considered complementary strategies rather than direct Anxa2-targeted treatments. Similarly, nucleic acid delivery platforms may provide potential tools for selectively modifying Anxa2 expression or associated molecular networks, although challenges related to specificity, delivery efficiency, and safety remain unresolved (Najafi et al., 2024).

Despite its therapeutic potential, the multifunctional nature of Anxa2 represents a major challenge for clinical translation. Anxa2 is not exclusively a disease-promoting factor; rather, it participates in physiological processes including membrane repair, fibrinolysis, endothelial barrier maintenance, vesicular trafficking, and regulation of inflammatory homeostasis. Therefore, indiscriminate systemic inhibition of Anxa2 may interfere with essential cellular functions and potentially result in undesirable toxicity. This concern is particularly relevant in the nervous and vascular systems, where Anxa2 contributes to tissue repair, neurovascular stability, and cellular stress adaptation. Accordingly, future therapeutic strategies should not aim at complete elimination of Anxa2 activity, but rather at selectively modulating disease-associated Anxa2 functions, such as abnormal protein interactions, pathological post-translational modifications, or context-specific signaling outputs. Approaches integrating cell-type-specific delivery systems, molecular interface targeting, and disease-stage-dependent intervention may provide safer alternatives to conventional broad inhibition strategies.

Overall, Anxa2 represents a biologically attractive but complex therapeutic target in neurological disorders. Current evidence supports its involvement in neurovascular repair, inflammatory regulation, and neuronal homeostasis; however, clinical translation requires a shift from broad inhibition toward precision modulation of disease-associated Anxa2 functions. Future studies integrating single-cell analysis, spatial multiomics, patient-derived models, and mechanistically designed clinical cohorts will be essential for determining whether Anxa2 can be effectively exploited as a biomarker or therapeutic target in neurological diseases.

## 6 Summary and future perspectives

Anxa2 is a multifunctional Ca^2+^-dependent phospholipid-binding protein involved in membrane organization, cytoskeletal remodeling, vesicular trafficking, fibrinolysis, and cellular stress responses. Across neurological models, changes in Anxa2 expression or activity have been associated with PI3K/AKT, MAPK/ERK, NF-κB, and TGF-β-related signaling. The strength of these associations, however, varies substantially among experimental systems. In many cases, current evidence does not distinguish direct pathway regulation from secondary effects caused by altered membrane trafficking, receptor organization, protein handling, or cellular homeostasis. Anxa2 should therefore be viewed less as a universal upstream regulator of canonical signaling pathways and more as a membrane-associated scaffold whose molecular interactions can reshape multiple cellular processes.

This distinction is important when interpreting the apparently divergent effects of Anxa2 across neurological diseases. Differences among neuronal, glial, endothelial, immune, and tumor models may reflect genuine cell-specific functions, but they may also arise from differences in experimental design, disease stage, subcellular localization, perturbation strategy, or compensatory cellular responses. Thus, “context dependence” should remain a testable hypothesis rather than a general explanation for conflicting findings. Establishing whether Anxa2 has distinct functions in different biological settings will require direct comparison of these variables within well-controlled experimental systems.

A central unresolved question is what determines the direction of Anxa2-associated effects. Current evidence does not support a simple protective-versus-pathogenic dichotomy. Instead, Anxa2 function is likely shaped by cell identity, intracellular versus extracellular localization, post-translational modifications, interacting partners, and the duration of perturbation. These factors may influence membrane trafficking, receptor organization, fibrinolysis, inflammatory signaling, and protein homeostasis, thereby producing different downstream phenotypes. Defining which of these determinants are causal will be necessary before stage-dependent or disease-specific roles can be assigned to Anxa2 with confidence.

Future studies should therefore move beyond expression-based associations and prolonged gain- or loss-of-function models. Single-cell and spatial profiling can identify where Anxa2-associated cellular states occur, but causal mechanisms will require cell-type-specific and temporally controlled manipulation, acute protein depletion, and rescue experiments using localization- or interaction-selective Anxa2 mutants. Validation at endogenous protein levels and direct analysis of relevant protein–protein interfaces will also be important. Such approaches should help separate Anxa2-specific molecular effects from secondary changes in membrane organization, trafficking, or chronic cellular adaptation.

These mechanistic distinctions are equally important for therapeutic development. Because Anxa2 contributes to membrane repair, endothelial homeostasis, fibrinolysis, vesicular trafficking, and inflammatory regulation, broad systemic inhibition is unlikely to be appropriate across neurological diseases. A more plausible strategy is to modulate disease-associated Anxa2 functions within defined cell populations and therapeutic windows. Depending on the disease context, this may involve targeting extracellular Anxa2 complexes, abnormal post-translational states, or specific protein–protein interaction interfaces rather than altering total Anxa2 abundance. Candidate interventions should therefore be evaluated not only for efficacy, but also for cell specificity, target engagement, and preservation of physiological Anxa2 functions.

Clinical translation will also require a clearer connection between mechanism, patient heterogeneity, and therapeutic response. Biomarker studies should validate Anxa2-related signatures in well-characterized and independent patient cohorts to determine whether they provide meaningful information on disease state, prognosis, or treatment response. Therapeutic studies should use patient-derived and disease-relevant models to identify the settings in which Anxa2 modulation is most likely to be beneficial. Single-cell analysis, spatial multiomics, structural approaches, and targeted delivery technologies may support this process, but they should be used to address defined biological or translational questions rather than as ends in themselves. Similarly, emerging cell- or nucleic acid-based delivery platforms may provide useful tools for selective modulation, although their direct relationship with Anxa2 biology requires further validation.

Overall, current evidence is more consistent with Anxa2 acting as a multifunctional membrane-associated scaffold and interaction hub than as a uniform upstream regulator of multiple signaling pathways. The next phase of research should therefore move from pathway-level associations toward causal definition of Anxa2 interaction modules at endogenous protein levels, followed by validation in disease-relevant and human-derived systems. This framework should clarify when Anxa2 can serve as a biomarker, when it is therapeutically actionable, and when intervention may disrupt essential physiological functions. For neurological disease, precision modulation of defined Anxa2 functions, rather than global suppression, provides the most rational path toward clinically meaningful translation.
